# Multimodal data on bipedal locomotion during prolonged treadmill recordings at varying speeds

**DOI:** 10.1038/s41597-026-07445-3

**Published:** 2026-05-20

**Authors:** Dominik Krumm, Daniel Koska, Jayant Wakode, Stephan Odenwald, Christian Maiwald

**Affiliations:** 1 Chemnitz University of Technology, Sports Equipment and Technology, Chemnitz, Germany; 2 Chemnitz University of Technology, Professorship of Research Methodology and Data Analysis in Biomechanics, Chemnitz, Germany; 3 University of Münster, Münster, Germany

## Abstract

This dataset provides curated multimodal recordings of human bipedal locomotion collected from 18 healthy adults during prolonged treadmill activity at four controlled speeds (4 km ∙ h^−1^, 6 km ∙ h^−1^, 9 km ∙ h^−1^, and 12 km ∙ h^−1^). Each participant completed three continuous treadmill trials comprising multiple speed intervals, totalling approximately forty minutes of recorded activity. Kinematic data were captured using an optical motion capture system with a full-body marker setup. Additional movement-related signals were recorded using inertial measurement units attached to the sacral crest and to the lateral side of each shoe. These units provided acceleration and angular velocity data. Kinetic data were recorded simultaneously from dual-belt treadmill force plates, and plantar pressure data were recorded using pressure-sensing insoles. The resulting dataset provides synchronised, multimodal data from all systems, including detailed marker and force trajectories, as well as pressure, acceleration and angular velocity signals. Vicon processing pipelines and MATLAB scripts are provided to ensure full reproducibility. The dataset adheres to FAIR principles and supports a range of biomechanical and movement science applications.

## Background & Summary

The present study was designed as a cross-sectional investigation of human bipedal locomotion. It incorporated repeated measurements from the same participants under four different speed conditions. This study design enables a descriptive characterisation of movement behaviour, which may be used to generate new hypotheses regarding locomotor mechanics, sensor validation, or adaptation phenomena. In conducting this work, we pursued three primary objectives. First, we aim to determine the mechanical power output during bipedal locomotion by applying the analytical model of Jenny and Jenny^1^ to experimentally measured ground reaction forces obtained from an instrumented treadmill. This objective is intended to test the assumptions made by Jenny and Jenny^1^ in their analytical model of mechanical power during human running. Second, we seek to evaluate the validity of a body-attached power meter. Wearable sensors, including plantar pressure insoles and inertial measurement units, are used together to estimate ground reaction forces. These estimated forces are then processed through the same analytical model to calculate mechanical power, which will be compared with the reference values. Third, we aimed to establish and share a multimodal research dataset on bipedal locomotion that adheres to the FAIR data principles (Findable, Accessible, Interoperable and Reusable)^2^. This openly available dataset is expected to support a range of investigations into human movement mechanics and contribute to reproducible research in biomechanics.

Several openly available datasets have made a significant contribution to the field of human movement science. Notable examples include datasets on lower-limb kinematics and kinetics during continuously varying locomotion^3^, gait biomechanics of above-knee amputees^4^, multimodal data from walking and stair tasks^5^, optical motion capture of overarm throwing^6^, movement techniques of Kyokushin karate athletes^7^ and an Asian-centric dataset of activities of daily living^8^. While these datasets provide valuable biomechanical information, they generally lack long-duration treadmill recordings and frequent speed transitions within a single trial. In recent years, larger and more comprehensive datasets have emerged, such as the Gait120 dataset^9^, which provides full-body kinematics and electromyography for 120 healthy adults; a dataset on walking and running biomechanics across varying step widths^10^; and a normative kinematic dataset of healthy adults performing gait and sit-to-stand movements^11^. While these datasets enrich the field, they typically do not include extended-duration treadmill measurements with multimodal synchronisation across kinetic, kinematic and wearable sensor data.

Our dataset has already been utilised by recent research for new investigations. Weidensager *et al*. employed the data to estimate vertical ground reaction forces from plantar pressure through high-dimensional approximation^12^; Sanseverino *et al*. devised techniques to estimate hiking events and temporal gait parameters using body-attached sensors^13^; and Krumm *et al*. conducted preliminary analyses on mechanical power estimation during running based on kinetic data^14^. These applications demonstrate the dataset’s versatility and its contribution to ongoing advancements in biomechanics and sports engineering.

## Methods

### Sample size planning

Following Bland & Altman’s recommendations for method-comparison studies^15^, the sample size was based on the expected precision of the Limits of Agreement (LoA). Assuming no relevant systematic bias, pilot data collected in preparation for the study were used to estimate the standard deviation of paired differences in joint-angle variables, which was set to 2.5° for planning purposes. This estimate was then used, together with an assumed tolerable uncertainty of 2° around each estimated LoA, to estimate the required sample size using the large-sample approximation described by Bland and Altman^16^. Under this approximation, the confidence interval around each estimated LoA is approximately $1.96\times {SD}\times \sqrt{3/n}$. This yielded a required sample size of approximately 18 participants; this was rounded up to 20 to allow for a small margin in planning. As data collection progressed, the estimated LoA and their confidence intervals were also inspected descriptively, with no notable changes in interval width observed beyond *N* > 10.

### Participants and ethics statement

The study was approved by the Ethics Committee of Chemnitz University of Technology (reference #101525731) and conducted in accordance with the Declaration of Helsinki. Written informed consent was obtained from all participants prior to data collection, including permission for data use in research and potential publication of anonymised results. Eligible participants were healthy, recreational adults aged 18–50 years with European shoe sizes between 36 and 47. Exclusion criteria included any acute or recent (within six months) lower-limb injury, medical conditions impairing locomotion, inability to provide valid proof of vaccination or a negative COVID-19 test, non-compliance with institutional hygiene policies, or suspected COVID-19 infection.

Eighteen participants (5 female, 13 male) met the inclusion criteria and provided written informed consent. Female participants were aged 24.8 ± 1.9 years (mean ± SD), with height 168.6 ± 6.8 cm, body mass 66.4 ± 10.3 kg, and BMI 23.2 ± 2.4 kg ∙ m^−2^. Male participants were aged 31.3 ± 7.3 years, with height 182.6 ± 6.7 cm, body mass 78.0 ± 11.7 kg, and BMI 23.3 ± 2.3 kg ∙ m^−2^. Individual subject metrics are provided within the publicly available dataset (see Data Records section for details). No personally identifiable information is included in the released dataset; all shared metadata are fully pseudonymised in compliance with privacy standards.

### Equipment

**Fig. 1:**
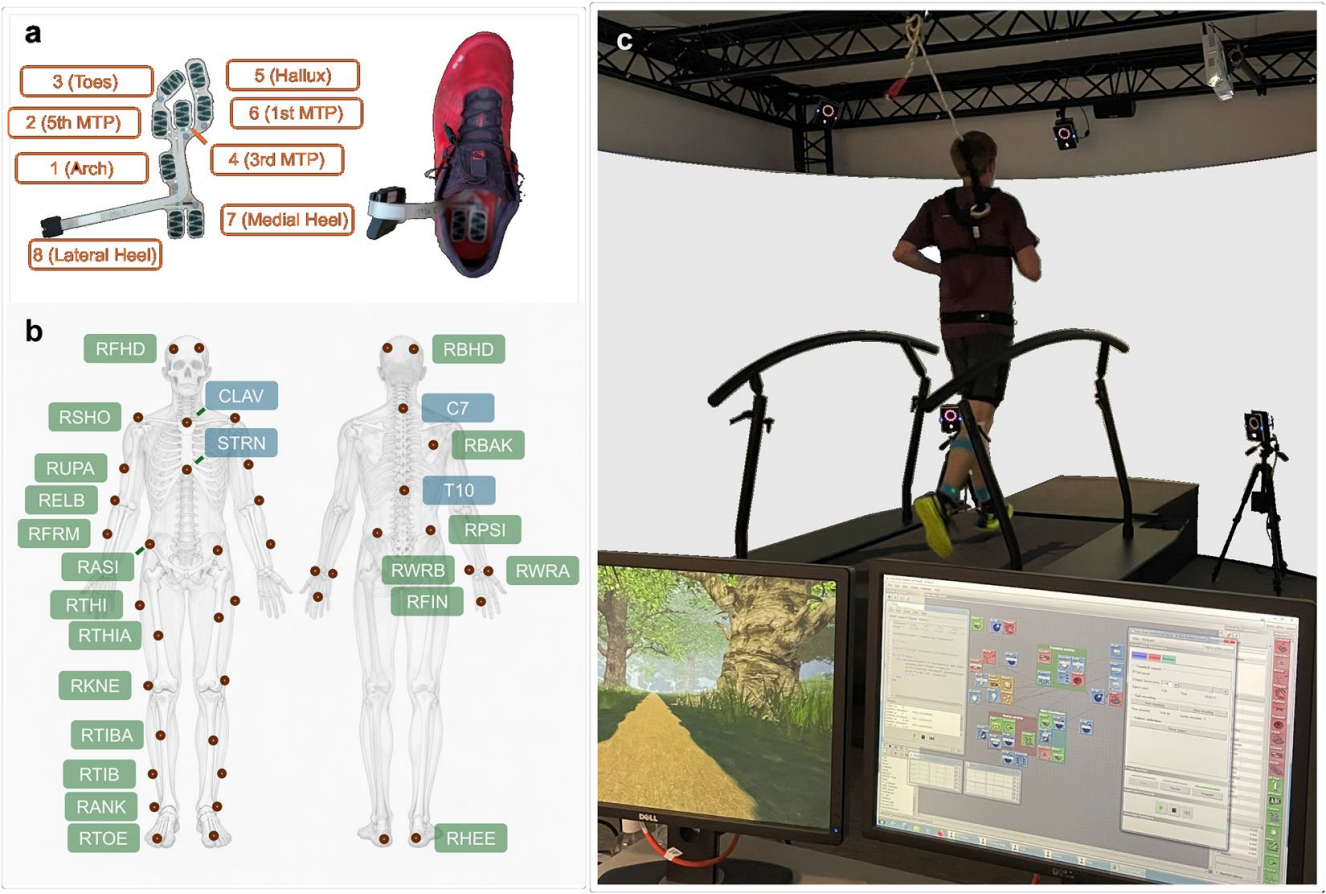
Experimental setup for instrumented treadmill-based gait analysis. (**a**) Pressure-sensing insole with eight embedded sensors, connecting electronics for wearable data acquisition, and a running shoe with the insole inserted. (**b**) Placement and labelling of 43 markers according to the Plug-In Gait FullBody Ai Functional protocol. (**c**) The GRAIL laboratory environment, featuring a dual-belt treadmill with integrated force plates, an optical motion capture system, and a 240° projection screen for immersive feedback. The participant provided consent for the publication of the image.

Data collection was performed using the Gait Real-time Analysis Interactive Lab (GRAIL) system (Motek Medical B.V., Houten, Netherlands), three body-attached sensor networks (BASNs; hereafter referred to as inertial measurement units – IMUs) (ENVISIBLE Sensors, Steinbeis-Forschungszentrum, Chemnitz, Germany), and a pair of pressure-sensing insoles (Smart Footwear Sensors HD-002, IEE, Echternach, Luxembourg). Each IMU comprised a compact, lightweight sensor node integrating tri-axial accelerometers and gyroscopes^17^. The pressure insoles were connected to the sensor nodes via plug-in connectors (Fig. 1a) and operated using the Envisible software suite. Participants wore their own neutral road running shoes without carbon plates or motion control features. The shoes were fitted with pressure insoles in the appropriate size. The insole size assigned to each participant (S, M, L, XL) is reported in the tab-separated file *IndividualSubjectMetrics.txt*, available under https://osf.io/dtqev. The physical dimensions of the insoles were: S (217.57 × 73.82 mm, EU 36–38), M (231.85 × 85.12 mm, EU 39–41), L (247.49 × 85.04 mm, EU 42–44), and XL (269.91 × 89.15 mm, EU 45–47).

The GRAIL setup (Fig. 1c) included a dual-belt treadmill with integrated force plates (M-Gait, Motek Medical B.V.) mounted on a two-degree-of-freedom motion platform, a fall-protection harness system, and a 240° cylindrical projection screen with wide-angle projectors, which remained blank throughout data collection. The optical motion capture system comprised ten infrared cameras (Vantage 5, Oxford Metrics, Oxford, UK) controlled via *Vicon Nexus 2* (version 2.12) and the *D-Flow* operator software.

### Procedure

Participants wore tight-fitting sports clothing and a safety harness connected to the treadmill’s overhead protection system. Pressure insoles were inserted beneath the inner soles of the participant’s athletic shoes. Two IMUs were attached laterally to each shoe, and one was affixed to the sacral crest using adhesive tape.

A biomechanist (≥5 years of experience) and certified GRAIL operator performed anthropometric measurements and attached 43 reflective markers (10 mm diameter) to predefined anatomical landmarks according to the Vicon Plug-in Gait Full Body Ai Functional model (Fig. 1b). Marker placement was performed by anatomical palpation and remained consistent across all measurement sessions. The marker set supports both the *Dynamic Plug-in Gait Model*^18^—Vicon’s implementation of the Conventional Gait Model^19,20^—and the *open MATLAB-based Plug-in Gait model* using functional joint centres determined by optimisation algorithms^21,22^. Angular conventions followed the *Vicon Nexus 2* user guide^18^. Any markers or IMUs that detached during a trial were reattached by the biomechanist whenever feasible, using the pre-marked anatomical locations. Reattachment was performed either during planned rest periods or while the treadmill was still running. In this case, participants briefly stepped onto the stationary side platforms (outside the running belts) to allow rapid reattachment using fresh adhesive tape. Such events were rare and resolved quickly. They are not explicitly annotated in the dataset.

After verifying sensor functionality and zeroing the treadmill’s force plates, participants stepped onto the treadmill and performed a brief static calibration (approximately 1s). Once marker labelling and model calibration were complete, participants performed a functional calibration (Range of Motion, ROM) trial involving controlled joint movements to define individual joint centres.

The dynamic recordings comprised three treadmill trials for each participant, totalling approximately 40 minutes of human bipedal locomotion activity. Trials varied in duration and activity sequence to systematically assess locomotion under different speed conditions. Trial 1 began with a 2-minute warm-up at 3 km ∙ h^−1^, followed by a 1-minute rest, and then three consecutive 3-minute activities—walking at 4 km ∙ h^−1^, hiking at 6 km ∙ h^−1^, and running at 9 km ∙ h^−1^—ending with a final 1-minute break (total duration: 13 minutes). Trial 2 lasted 11 minutes, and Trial 3 lasted 16 minutes; both included alternating walking, hiking, and running intervals with rest phases. To ensure comparable fatigue states across participants, the sequence and duration of activities were deliberately not randomised across trials, with ≥1-minute rest phases balancing order effects. Details of the exact sequences, including speed, timing, and rest intervals, are provided in Table 1. All participants except one (G14) ran at 12 km ∙ h^−1^ (see Technical Validation for details). Participants were given at least 1 minute of rest between trials.

**Table 1:** Summary of treadmill-based experimental protocol.

Trial	Treadmill speed		Elapsed Time	Duration	Activity	Frame	Labelling
	km/h	m/s	hh:mm	min			
1	3	0.83	00:00	2	Warm Up	1–30000	XXn01warmup
1	0	0	00:02	1	N pose (10 s) & Pause	30001–45000	XXn02pause
1	4	1.11	00:03	3	Walk A	45001–90000	XXn03walkA
1	6	1.67	00:06	3	Hike A	90001–135000	XXn04hikeA
1	9	2.5	00:09	3	Run A	135001–180000	XXn05runA
1	0	0	00:12	1	Pause	180001–195000	XXn06pause
**Total duration**			**00:13**				
2	0	0	00:00	1	Pause	1–15000	XXn07pause
2	9	2.5	00:01	3	Run B	15001–60000	XXn08runB
2	6	1.67	00:04	3	Hike A	60001–105000	XXn09hikeB
2	4	1.11	00:07	3	Walk B	105001–150000	XXn10walkB
2	0	0	00:10	1	Pause	150001–165000	XXn11pause
**Total duration**			**00:11**				
3	0	0	00:00	1	Pause	1–15000	XXn12pause
3	4	1.11	00:01	3	Walk C	15001–60000	XXn13walkC
3	9	2.5	00:04	3	Run C	60001–105000	XXn14runC
3	0	0	00:07	1	Pause	105001–120000	XXn15pause
3	6	1.67	00:08	3	Hike C	120001–165000	XXn16hikeC
3	12	3.33	00:11	3	Run Fast A	165001–210000	XXn17fastrunA
3	3	0.83	00:14	2	Cool Down	210001–240000	XXn18cooldown
**Total duration**			**00:16**				

Details of session/trial structure, treadmill speeds, ordered activity and pause sequences, elapsed times, durations, frame counts, and labelling conventions for all measurement phases and rest intervals.

Sampling frequencies were primarily determined by the technical capabilities of the respective devices. The insole pressure sensors were sampled at 100 Hz, the IMUs at 1000 Hz, the motion capture at 250 Hz, and the treadmill force plates at 1000 Hz. The force plate sampling rate was reduced from higher available frequencies, as 1000 Hz was considered sufficient for capturing ground reaction forces at the maximum running speed of 12 km ∙ h^−1^. IMU measurement ranges varied by trial: acceleration ±2 g, ±8 g or ±16 g; and angular velocity ±250 °/s, ±500 °/s, ±1000 °/s or ±2000 °/s. The exact configuration for each recording is provided in the tab-separated file *IMUSettingsPerTrial.txt*, available under https://osf.io/vq8ja. The insole pressure sensors had a measurement range of 250 mbar to 7 bar. The motion capture system and treadmill force plates were hardware-synchronised. The three IMUs were synchronised internally through a wireless server-client network. Owing to technical constraints, no direct cross-system synchronisation was available between laboratory-based systems and the wearable IMUs.

To ensure temporal alignment, each system was manually triggered at the start of each recording trial. This manual triggering typically resulted in a maximal time offset of 4 ms (i.e. within one motion capture frame at 250 Hz). The dataset is provided with these original, manually-aligned timestamps, which may contain small offsets due to manual triggering. Users requiring more precise synchronisation can apply event-based methods, such as those described by Weidensager *et al*., which align data streams based on physical events, like initial contact, in downstream analyses to minimise offset effects^12^. Corresponding event annotations are provided within the dataset to support such alignment procedures.

### Data processing

Data processing was conducted using a sequence of custom Vicon post-processing pipelines included in the public repository (Table 2). Static calibration trials (*cal01*) were processed using the 1_static pipeline in *Vicon Nexus*, which performed trajectory reconstruction, marker autolabeling, subject scaling, and model calibration. The resulting C3D and VSK files established the anatomical reference for subsequent analyses.

**Table 2:** Data processing pipeline for Vicon motion capture files.

Step (filename)	Input files	Pipeline name & stages	Output files
Calibration measurement (e.g. cal01)	HISTORY| SYSTEM| TRIAL.ENF |X1D|X2D|XCP	1_static → reconstruct, autolabel static, scale subject, static skeleton calibration, OCST calibration, Static Plug-in Gait processing, save trial	C3D| VSK
Range of Motion measurement (e.g. rom01)	HISTORY| SYSTEM| TRIAL.ENF |X1D|X2D|XCP	2_rom → combined processing, autolabel, gap filling, kinematic fit, woltring filter, OCST processing, SCORE calibration, functional skeleton calibration, save trial	C3D| VSK
Trial processing (e.g. run01)	HISTORY| SYSTEM| TRIAL.ENF |X1D|X2D|XCP	3a_reconstruct → combined processing, save trial	C3D
Export to MATLAB (e.g. run01)	C3D	3b_save2mat → run matlab operation	MAT
Segmentation (e.g. run01 → *n01)	C3D	4a_export_trials → export C3D	C3D
Activity labelling (e.g. *n01)	C3D	4b_label → label, save trial	C3D
Dynamic trial processing (e.g. *n01)	C3D	4c_dynamic → autolabel, gaps filling, woltring filter, kinematic fit, OCST processing, SCORE calibration, save trial	C3D
Export to MATLAB (e.g. *n01)	C3D	4d_save2mat → run matlab operation	MAT
Event detection & force filtering (e.g. *n01)	C3D	4e_getevents_filterGRF → run matlab operation	C3D
Dynamic Plug-in Gait Model (e.g. *n01)	C3D	5_dynamic_gait_model → detect events from forceplate, autocorrelated events, calculate gait cycle parameters, Dynamic Plug-in Gait, save trial	C3D

Outline of the custom pipeline steps, specifying input and output file types, processing parameters, and file functions. Formats include: ENF for database, SYSTEM for configuration, XCP for camera calibration, X1D and X2D for respective analogue and optical data, C3D for 3D motion trajectories, VST for skeleton templates, and VSK for kinematic subject models.

ROM trials (*rom01*) were processed with the 2_rom pipeline, which executed segmental autolabeling, gap filling, kinematic fitting, and anatomical segment calibration. The resulting joint range definitions serve as reference parameters for motion analysis.

Dynamic trials (*trial01–trial03*) were first reconstructed with the 3a_reconstruct pipeline to produce initial C3D files containing raw trajectories. The 3b_save2mat pipeline then converted these data into MATLAB format for further processing. Each trial was subdivided into activity-specific segments and renamed using the 4a_export_trials pipeline (e.g., *G01n01warmup*) following the conventions detailed in Table 1. Marker labelling consistency was checked using the 4b_label pipeline, and refined reconstruction, gap filling, and recalibration were performed using 4c_dynamic. Final files were exported in MATLAB format using the 4d_save2mat pipeline.

**Fig. 2:**
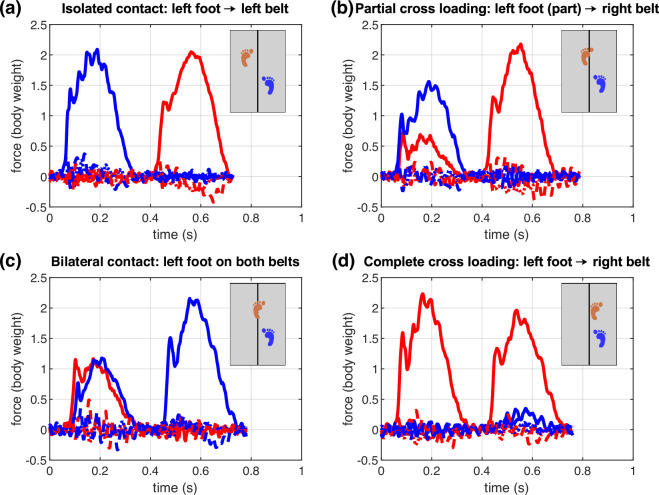
Ground reaction force scenarios on a dual-belt treadmill. (**a**) Isolated contact of the left foot on the left belt. (**b**) Partial cross-loading, with part of the left foot over the right belt. (**c**) Bilateral contact, where left foot spans both belts. (**d**) Complete cross-loading, with the left foot contacting only the right belt. For each scenario, vertical (solid), anterior–posterior (dotted), and medio-lateral (dashed) ground reaction forces are shown in body weight units. Red lines represent forces from the left plate and blue lines from the right plate.

Gait events, such as initial foot contact and foot off, were identified using the 4e_getevents_filterGRF pipeline. This pipeline was developed specifically to handle cases in which a foot partially steps onto the opposite treadmill belt (Fig. 2). Events were detected using heel and toe marker trajectories. Local minima and inflection points were identified by applying adaptive stride-specific thresholds. The accompanying MATLAB script, getEventsBasedonFootMarkers.m, implements this algorithm using only kinematic marker data and treadmill speed. The detected events were automatically reinserted into the corresponding *Vicon Nexus* trial files. This approach provides identification of gait-events, even under challenging cross-loading conditions. Centralised event lists derived from foot markers and pressure insoles using the accompanying MATLAB script ExtractPressureInsoleEvents.m are provided within the dataset.

## Data Records

The datasets described in this Data Descriptor are publicly available via the Open Science Framework (OSF) and are distributed across three DOI-registered components. The main project contains metadata, documentation, codes, pipelines, and example results (10.17605/OSF.IO/KCQTF)^23^. Processed data are provided as aggregated, participant-level ZIP archives (10.17605/OSF.IO/N86G7), as are the raw data (10.17605/OSF.IO/G79VH). All files are provided in open, non-proprietary formats to ensure long-term accessibility and interoperability.

The dataset contains raw and processed biomechanical recordings from 18 healthy participants who completed three treadmill trials comprising bipedal locomotion activities at multiple controlled speed conditions. Each participant completed three trials, totalling approximately 40 minutes of recorded activity.

For most use cases, the processed and raw data components provide the primary access points, as they contain curated participant-level download packages without requiring navigation of the full repository structure. The complete dataset can be reconstructed by combining the contents of the main project with all ZIP archives from the processed and raw data components.

The aggregated packages follow consistent naming conventions:

- Processed data: *ldbl_treadmill_processed_[mat|vicon]_subj_[ID]_v01*
- Raw data: *ldbl_treadmill_raw_[insole|vicon]_subj_[ID]_v01*

The modality tag specifies the data type, *[ID]* denotes the participant identifier, and *v01* indicates the version.

The dataset^23^ is organised into six main components on OSF that support two complementary access modes: Aggregated downloads via two packages (*01_Processed_data_download* and *02_Raw_data_download*) for direct data extraction and rapid reuse. Full repository access via four additional components (*Data*, *Pipelines*, *Codes*, and *Results*) to preserve the original hierarchy, metadata and complete workflows for replication and custom processing.

Within the main project, the repository is organised into components that preserve the full data hierarchy and processing workflow. The *Data* component contains all raw and processed recordings organised by participant (e.g., S03, G01–G17). Each participant folder includes two directories: *Raw_data* and *Processed_data*. The *Raw_data* directory contains pressure insole recordings stored as ENVISIBLE text files with comma-separated structure, as well as a *Vicon* subdirectory containing motion capture system files and calibration data. Filenames correspond to the original acquisition naming conventions and were preserved unchanged to maintain traceability with the raw recordings. The *Processed_data* directory is divided into *Mat* and *Vicon* subdirectories containing processed datasets and analysis outputs. These include MATLAB-compatible MAT files for each trial and activity, together with extracted joint-angle parameters and event-based temporal features.

The *Pipelines* component contains the Vicon Nexus pipeline files used for automated data processing. These pipelines cover static calibration, range-of-motion trials, marker reconstruction, dynamic modelling, labelling, event detection, and data export (1_Static.Pipeline, 2_rom.Pipeline, 3a_reconstruct.Pipeline, 3b_save2mat.Pipeline, 4a_export_trials.Pipeline, 4b_label.Pipeline, 4c_dynamic.Pipeline, 4d_save2mat.Pipeline, 4e_getevents_filterGRF.Pipeline, and 5_dynamic_gait_model.Pipeline). Each pipeline file is annotated to indicate its role within the data processing workflow.

The *Codes* component contains custom MATLAB scripts used for additional data processing and analysis. These scripts are distributed under the ISC License and include converters (SaveGrailDatatoMAT.m, c3d2mat.m), data import and processing tools (ImportEnvisible.m, LoadForceData.m, LoadPressureData.m, ProcessForceData.m, ProcessPressureInsoleData.m), parameter extraction utilities (GetTemporalParameters.m, GetSegmentedTemporalParameters.m), and visualisation scripts (PlotBAPs4GRAIL.m, PlotGaitCyclesHikeParameters4GRAIL.m, PlotGaitCyclesHikeParameters4HikeEvents.m). Higher-level batch processing and event-detection scripts such as EvaluateLabExperiment.m are also provided. The *DynamicPiG.m* script included with Vicon Nexus (Plug-In Gait, AGW) is referenced for use in the dynamic gait modelling pipeline.

**Fig. 3:**
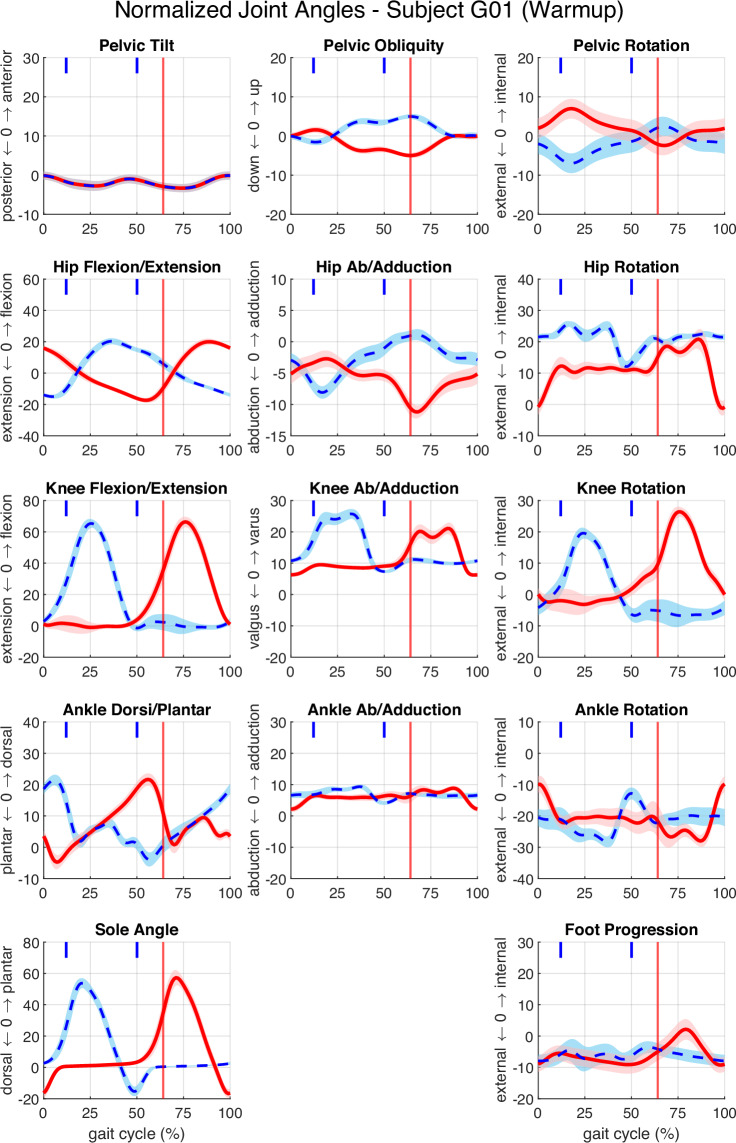
Lower-limb joint angle trajectories during warm-up (3 km ∙ h^−1^) for a single subject. Mean and standard deviation (SD) of left and right joint angles across the gait cycle. Left-side means are shown as thick red lines with shaded SD bands; right-side means are shown as dashed blue lines with shaded SD bands. The vertical red line marks left foot off, and short blue vertical lines denote right foot events. Columns correspond to sagittal, frontal, and transverse plane; rows display pelvis, hip, knee, ankle, and foot joints. Panel titles specify the joint and anatomical plane (e.g., “Pelvic Tilt”).

**Fig. 4:**
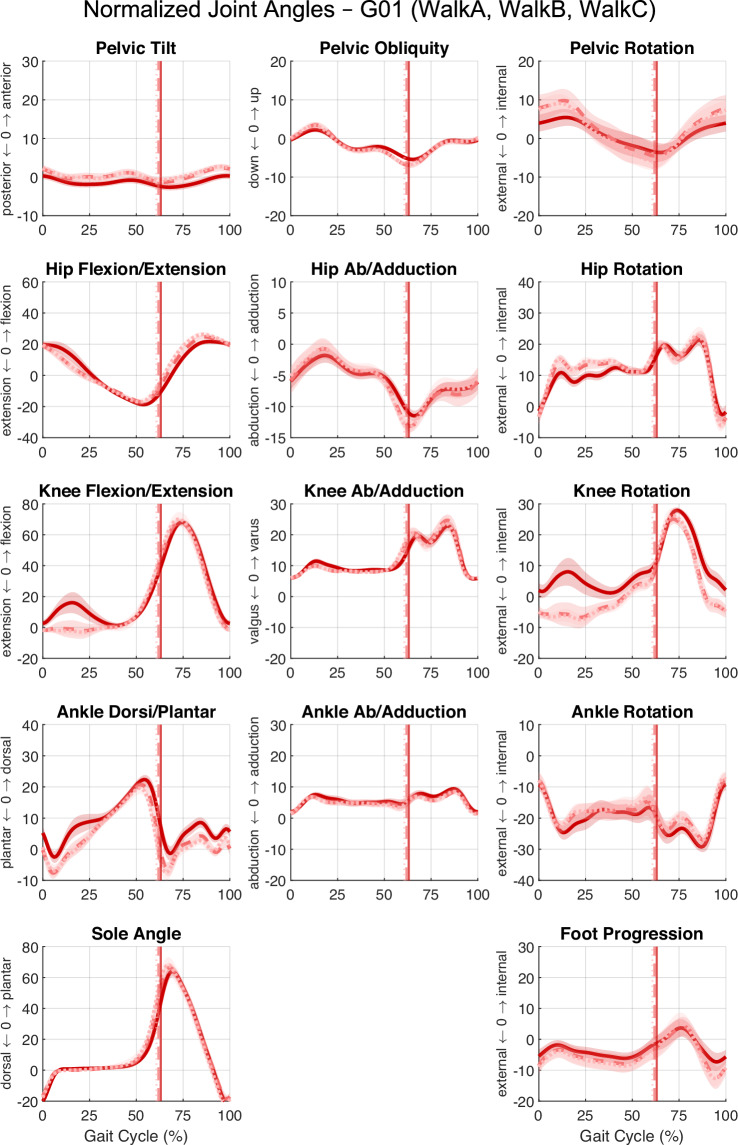
Lower-limb joint angles from three walking (4 km ∙ h^−1^) trials for a single subject. Overlay of mean and standard deviation (SD) for left-side joint angles from three trials: walkA (solid bright red), walkB (dashed medium red), and walkC (dotted dark red). Shaded regions indicate SD for each trial. Vertical lines mark stance phase termination (left foot off) for each trial. Panel layout and anatomical labelling are identical to Fig. 3.

The *Results* component contains example figures and output files derived from related publications by Sanseverino *et al*., including Fig. 2_BAPs.svg, Fig. 3_allactivities.svg, and Fig. 4_segmentedactivities.svg^13^. These files can be used to replicate published results, verify workflow correctness, or serve as templates for new analyses.

Metadata and supporting files are provided in the main project. The tab-separated file entitled *IndividualSubjectMetrics.txt* provides individual subject metrics for each participant, including sex, age, height, body mass, BMI, and pressure insole size. As the IMUs were configured with differing measurement ranges during the data collection process, the applied accelerometer and gyroscope ranges for each recording are documented in the file entitled *IMUSettingsPerTrial.txt*. The foot contact events, derived from foot markers during motion capture, are provided in the file designated *EventList_ViconGRF_AllTRials.csv*. Similarly, the foot contact events derived from pressure insole data are provided in the file designated *EventList_PressureInsole_AllTrials.csv*. The file *Download_links.md* provides an index of repository components and direct download locations. Note that the cohort comprises 13 males and 5 females, which limits the suitability for sex-stratified analyses.

Additional documentation describing the repository structure, dataset organisation, and download procedures is available in the OSF project Wiki. All pipelines and MATLAB scripts were tested for compatibility with Vicon Nexus 2.1.2 and MATLAB R2024a.

## Data Overview

To illustrate the dataset’s content and overall measurement quality, exemplary joint angle trajectories of the lower limb during a warm-up trial from a single participant are shown (Fig. 3). Overlayed joint angles from three consecutive walking trials at 4 km ∙ h^−1^ (Fig. 4) further demonstrate the intra-subject repeatability of locomotion patterns. The complete dataset includes joint kinematics for all lower and upper limb segments. Users seeking additional visualisation or summary statistics can generate them directly using the provided MATLAB scripts.

## Technical Validation

The technical quality of the dataset was evaluated using three core metrics for each recorded trial: (1) the number of unused markers, (2) the number of trajectory gaps, and (3) the percentage of successfully labelled markers out of a total of 43 markers. Across all sessions, the dataset demonstrated high technical integrity.

In the first trial alone, participants completed an average of 659 loading cycles, illustrating the high mechanical and operational demands placed on the measurement setup. All systems operated reliably throughout all trials, confirming stable performance under repeated locomotor loading.

The median number of unused markers was 0 (range: 0–2), indicating consistent marker placement and tracking quality. The median number of trajectory gaps was also 0 (range: 0–18), reflecting stable marker attachment and minimal signal loss. Nearly all measurements contained zero unused markers and zero gaps, with maximum observed values of two unused markers and 18 gaps.

Nearly all trials contained zero unused markers and zero gaps. Specifically, 251 out of 323 trials (78%) achieved complete labelling of all 43 markers. The mean proportion of labelled markers across all trials was 99.55 ± 1.12%. Even the trial with the lowest recorded value (90%) remained well within acceptable limits for biomechanical analysis, confirming the robustness and consistency of the dataset. One participant (G14) did not perform the 12 km ∙ h^−1^ run (fastrunA) due to safety concerns.

**Fig. 5:**
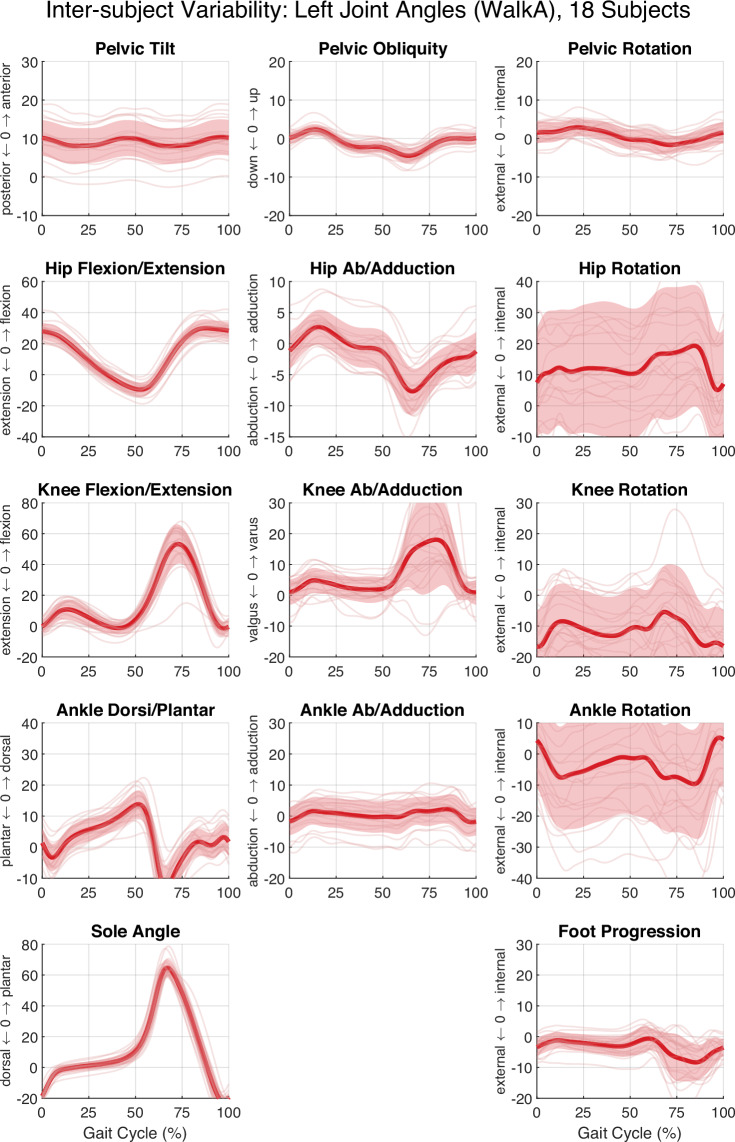
Inter-subject variability of left lower-limb joint angles during walking (4 km ∙ h^−1^). Overlay of mean joint angle trajectories for 18 subjects during walkA (thin coloured lines), with overall group mean (thick dark red line) and inter-subject standard deviation (SD; shaded area) for each joint across the gait cycle. Panel layout and anatomical labelling are identical to Fig. 3.

**Fig. 6:**
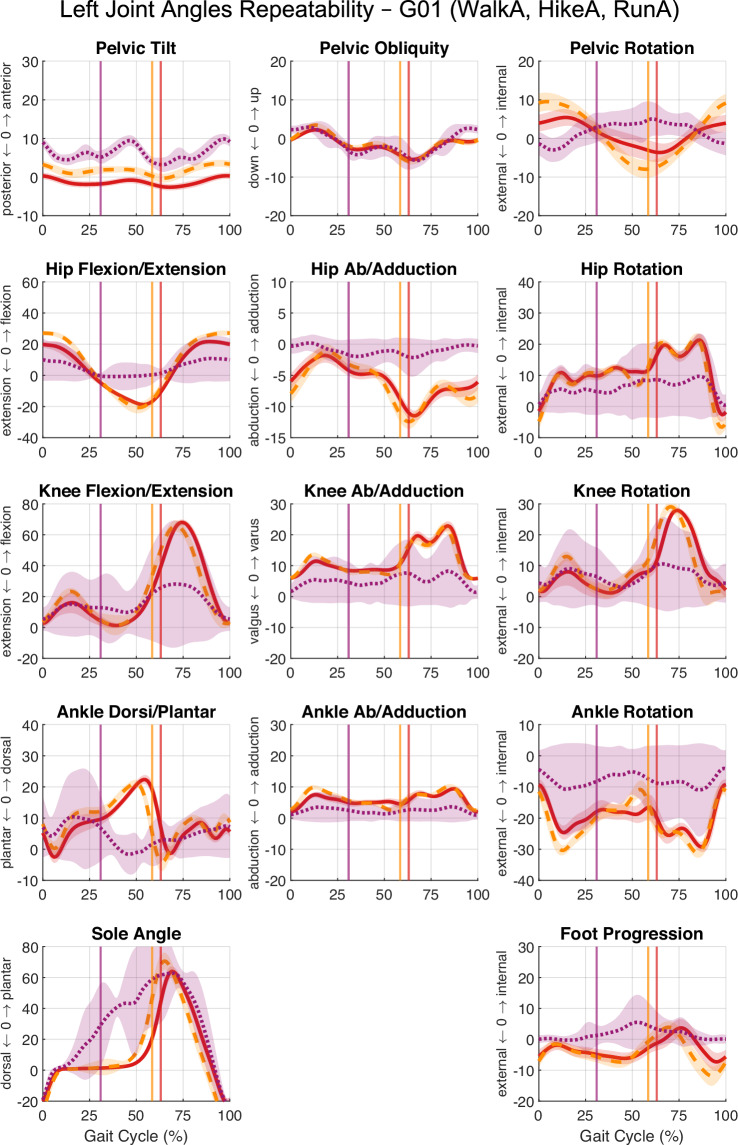
Left lower-limb joint angles across three locomotion modes within a single session. Overlay of mean and standard deviation (SD) for left-side joint angles from three modes: walkA (4 km ∙ h^−1^, solid bright red), hikeA (6 km ∙ h^−1^, dashed medium red), runA (9 km ∙ h^−1^, dotted dark red) for the same subject and trial. Shaded regions indicate SD for each mode. Vertical lines mark stance phase termination (left foot off) for each trial. Panel layout and anatomical labelling are identical to Fig. 3.

To illustrate data reliability and representativeness of locomotor kinematic patterns among healthy adults, Fig. 5 presents inter-subject variability of lower-limb joint angles during walking at 4 km ∙ h^−1^. The observed trajectories show consistent gait kinematics across participants. Additional examples of within-subject variability across locomotion modes are provided in Fig. 6. A detailed per-trial summary of all quality metrics is included in Table 3, enabling users to independently verify technical performance or filter trials according to specific inclusion criteria.

**Table 3:** Data quality summary of technical data quality metrics for each measurement in the dataset, including the number of unused markers, number of gaps, and percentage of markers successfully labelled.

Participant	Trials	Unused Markers	Gaps	Markers Labelled Mean ± SD	Non-perfect Trials	Abbreviated Name-ID of Non-perfect Trials (% labelled)
S03	18	1	1	99.06 ± 1.15	2	n05 (99), n06 (95)
G01	18	2	0	99.06 ± 1.90	6	n05 (98), n06 (92), n11 (99), n16 (98), n17 (98), n18 (98)
G02	18	0	10	99.61 ± 0.68	5	n07 (99), n14 (98), n15 (99), n17 (98), n18 (99)
G03	18	0	4	100.00 ± 0.00	3	n02 (100), n06 (100), n11 (100)
G04	18	3	4	99.44 ± 0.76	7	n05 (99), n06 (98), n07 (98), n08 (98), n09 (99), n15 (99)
G05	18	2	14	99.06 ± 1.43	7	n02 (99), n04 (99), n05 (96), n06 (99), n15 (97), n17 (96), n18 (97)
G06	18	0	13	99.61 ± 0.83	5	n02 (100), n06 (98), n12 (99), n15 (97), n17 (99)
G07	18	0	4	100.00 ± 0.00	3	n02 (100), n06 (100), n11 (100)
G08	18	0	11	99.50 ± 1.01	7	n02 (100), n05 (99), n06 (99), n11 (99), n14 (98), n15 (96)
G09	18	2	4	99.06 ± 1.31	8	n08 (99), n09 (99), n10 (98), n14 (99), n15 (98), n16 (98), n17 (96), n18 (96)
G10	18	0	1	99.89 ± 0.46	2	n02 (100), n14 (98)
G11	18	0	4	99.94 ± 0.23	2	n12 (100), n15 (99)
G12	18	0	21	99.22 ± 2.27	6	n05 (99), n06 (90), n11 (99), n15 (100), n17 (99), n18 (99)
G13	18	1	9	99.56 ± 0.76	6	n02 (100), n06 (99), n12 (98), n13 (99), n15 (98), n18 (98)
G14	17	1	23	99.18 ± 1.38	7	n06 (99), n09 (99), n11 (100), n14 (96), n15 (97), n16 (96), n18 (99)
G15	18	0	5	99.83 ± 0.69	1	n15 (97)
G16	18	0	11	99.61 ± 0.68	5	n02 (99), n04 (99), n05 (98), n06 (99), n15 (98)
G17	18	0	4	99.67 ± 0.67	5	n02 (100), n06 (98), n07 (98), n14 (99), n15 (99)

Participant-wise summary of marker-labelling quality across trials. Non-perfect trials indicate recordings with at least one unused marker, one gap, or a markers-labelled value below 100%. Trial abbreviations: n01…warmup, n02…pause, n03…walkA, n04…hikeA, n05…runA, n06…pause, n07…pause, n08…runB, n09…hikeB, n10…walkB, n11…pause, n12…pause, n13…walkC, n14…runC, n15…pause, n16…hikeC, n17…fastrunA, n18…cooldown. Participant G14 completed 17 trials because n17 was not performed.

## Usage Notes

The dataset is publicly accessible via three complementary OSF components: the main project (metadata, documentation, codes, pipelines, and example results), and two data components providing aggregated raw and processed data packages. Users seeking rapid access are advised to use the processed or raw data components, which provide participant-level ZIP archives organised by modality. Users requiring full reproducibility or custom processing should additionally access the main project, which contains the complete repository structure. To obtain the complete dataset, all ZIP archives from both data components must be downloaded alongside the files of the main project. Files can be downloaded individually or as ZIP archives through the OSF Files interface. Because OSF overview pages may display only partial file listings, users should navigate into individual components or folders to access the full contents. A complete index of download links is provided in *Download_links.md*. The dataset is released under the Creative Commons Attribution 4.0 International (CC BY 4.0) license. Users reusing the dataset should cite both the OSF resource (main project DOI) and this Data Descriptor.

## Data Availability

All data described in this Data Descriptor are openly available via the Open Science Framework (OSF) and are distributed across three DOI-registered components: • Main project: 10.17605/OSF.IO/KCQTF^23^ • Processed data: 10.17605/OSF.IO/N86G7 • Raw data: 10.17605/OSF.IO/G79VH The repository contains raw and processed biomechanical recordings of 18 healthy participants performing prolonged treadmill locomotion activities at four different speeds, with three trials per participant. All data are provided without access restrictions in open or widely supported formats to facilitate reuse and long-term accessibility.

## Code availability

All custom MATLAB scripts and Vicon Nexus pipelines used for data processing, analysis, and event detection are openly available within the *Codes* and *Pipelines* components of the OSF repository (https://osf.io/kcqtf^23^). Scripts are released under the ISC License, and all pipeline files are compatible with Vicon Nexus 2.1.2. No access restrictions apply.
